# Evaluation of Functional Impacts of Vision Impairment: Progress, Challenges and Future Directions

**DOI:** 10.1007/s44402-026-00121-4

**Published:** 2026-06-13

**Authors:** Joanne M. Wood, Alex A. Black, Andrew J. Kolarik, Matthew Timmis, Shahina Pardhan

**Affiliations:** 1 Centre for Vision and Eye Research, Optometry and Vision Science, Queensland University of Technology (QUT), Brisbane, Queensland Australia; 2 Vision and Eye Research Institute, School of Medicine, Anglia Ruskin University, Cambridge, UK; 3 School of Psychology, University of East Anglia, Norwich, UK; 4 Department of Psychology, Cambridge Hearing Group, University of Cambridge, Cambridge, UK; 5 Cambridge Centre for Sport and Exercise Sciences (CCSES), School of Psychology and Sport Science, Anglia Ruskin University, Cambridge, UK

**Keywords:** Audition, Driving, Falls, Mobility, Safety, Vision impairment

## Abstract

Vision impairment has profound consequences for everyday functioning and quality of life. This review summarises recent evidence on the impact of vision impairment in three functional domains: mobility and falls risk, driving, and the interaction between vision impairment and hearing. Individuals with vision impairment often adopt more cautious gait and visual search behaviours; however, they remain less stable and at increased falls risk, dependent on the type and severity of vision impairment. Wearable technologies enable continuous measurement of movement in naturalistic settings. Future research should leverage these tools to examine daily mobility patterns, fatigue and environmental exposure, and evaluate interventions in real-world contexts. Both central and peripheral vision play critical roles in driving. However, the impact of vision impairment on driving outcomes depends on the severity of loss as well as drivers’ insight into their vision impairment and adoption of behavioural and compensatory strategies. Future studies should incorporate naturalistic driving methods and advanced vehicle technologies to explore compensatory behaviours and interventions and their impact on real-world safety. Hearing abilities may improve or decline following vision loss, depending on the task. Musical perception and pitch discrimination are often enhanced in early-onset blindness, possibly reflecting recruitment of visual cortical areas by auditory processing, while performance on complex spatial hearing tasks can be poorer, as vision calibrates auditory spatial representations. Future research should determine how cross-modal plasticity impacts performance and explore how auditory training or multisensory rehabilitation may enhance functional adaptation. In summary, research highlights the importance of both central and peripheral vision and behavioural adaptations and compensatory strategies, with key challenges being the ability to measure real-world function and understand individual variability. Future work should integrate advanced assessment technologies and interdisciplinary approaches to better characterise functional outcomes and inform interventions to enable functional independence.

Key Points

- This paper provides a current review of the impact of vision impairment on mobility and falls, driving, and audition and future directions in these fields.
- Vision impairment results in reduced balance, gait adaptations and alters gaze behaviours, significantly increasing falls risk. Vision impairment also negatively affects driving performance, particularly hazard detection, with mixed evidence for crash risk due to compensatory behaviours. Blindness and vision impairment lead to both enhancements (e.g., sound localisation, echolocation, musical abilities) and impairments (e.g., spatial hearing, distance perception) in auditory function.
- Advances in technology provide opportunities for naturalistic research both in mobility (e.g., wearable technology) and driving (e.g., in-vehicle monitoring devices) assessments that inform interventions including training programmes and incorporating user-centred assistive technologies.

## Introduction

Understanding the functional impact of vision impairment is particularly important in an ageing demographic, given the associated rise in age-related vision impairment [1]. Research continues to explore the effect of different types of vision impairment on everyday functions, including mobility, falls, driving and the impact of vision loss on hearing, in order to develop evidence-based interventions to improve quality of life and independence. This review focuses on recent advances in the assessment of functional impacts in the domains of falls, balance and gait (or mobility), driving performance and safety, and audition, including methods of assessment, key recent evidence in the field, along with future directions for research.

## Vision Impairment and Mobility and Falls

The degradation in visual information in individuals with vision impairment impacts various parameters, including their balance, gait characteristics and gaze strategies while navigating the environment, and ultimately increases the risk of falls and subsequent injury. Assessment of balance comprises static tasks ranging from simple timed stand tests to more advanced force plate systems. Assessment of gait can include navigation through a ‘mobility course’ as individuals walk a set distance along a clear, unobstructed path, negotiate floor-based obstacles and/or surface height changes (steps or stairs), through to assessment in specialised gait labs with 3-D motion capture systems. Falls are typically recorded through self-reported retrospective falls or prospectively using falls diaries.

### Postural Control

Balance or postural control is fundamental to everyday functions, including transitional tasks such as sit-to-stand tasks and walking, including on uneven terrain and stair negotiation, and is controlled through input from the visual, vestibular and somatosensory systems. Of these, the visual system provides one of the most important sources of information [2]. The visual contribution to postural stability increases with age in order to compensate for age-related deteriorations in the somatosensory and vestibular systems [3]. Failure to adapt to the increasing reliance on vision with ageing results in reduced postural stability [4, 5], which can lead to an increased risk of falls [6–8]. The challenge for individuals with vision impairment is that they cannot compensate adequately for these age-related changes and therefore rely more on the somatosensory contribution for balance control with associated risk of falling [9–12].

Research highlights the association between reductions in visual function and postural instability, particularly when standing on foam, which disrupts the vestibular and somatosensory systems. Reduced visual acuity through either simulated optical blur or cataracts [2, 13, 14], or in cohorts with a range of vision impairments [15–21], has been shown to reduce postural stability. Similarly, impaired contrast sensitivity [19, 22, 23], visual field loss [24–27], and impaired visual motion perception [27–29] are associated with reduced postural stability.

Cataract has been associated with reduced postural stability in studies of simulated cataracts in older adults [13], as well as self-reported cataracts [16, 30], while cataract surgery has been associated with improvements in postural stability [31, 32].

Older adults with age-related macular degeneration (AMD) exhibit greater postural sway when standing on a foam surface under photopic [9, 26, 33] and mesopic conditions [34], with differences typically being greater for sway in the anterior-posterior than the medio-lateral direction. Reduced postural stability in AMD has been associated with reduced contrast sensitivity [9, 26] and increased falls risk [35], which increased in the presence of a secondary task [11].

Postural stability is also impaired in those with glaucoma on a range of balance tests [24, 36], and is associated with visual field loss, including binocular loss in the inferior field [24, 25] and loss of monocular fields and contrast sensitivity [36]. Individuals with glaucoma with more severe monocular or binocular field loss also had a lower visual contribution to postural sway than controls [10, 12].

### Gait Behaviours

Gait refers to the coordination of body movements to enable safe travel through the environment. Reductions in visual function may impact gait characteristics through reductions in postural control and balance confidence, as well as visually guided motor control required to avoid obstacles or negotiate steps and stairs. As a result, individuals with vision loss often adapt their gait behaviours. These adaptations may include taking longer to complete walking or stepping tasks (typically to increase planning time prior to entering the relatively unstable period of single support when stepping over an obstacle or up onto a step), swinging the foot slower and raising the foot higher to ensure greater clearance from the top of the obstacle or step edge, or increasing the double support time (time with both feet on the ground) to support balance. However, while these adaptations may reduce the likelihood of making contact with a step edge by raising the foot higher, or potentially increase the ability to recover if a step edge is hit by slowing the swinging foot, they can also lead to increased time in the relatively unstable period of single support that can increase medial-lateral sway [37], with the potential risk of sideways falls leading to hip fractures. Importantly, these adaptations are also context dependent, where gait is adjusted to different environmental features, such as avoiding obstacles or walking on uneven or slippery surfaces.

Early repeated measures studies showed adaptive gait changes resulting from simulated refractive blur. In particular, changes in gait characteristics have been reported when negotiating steps in the presence of both binocular and monocular refractive blur [37], but also when using different optical corrections such as multifocal spectacles [38, 39], or monovision contact lenses [40]. Some of these adaptive gait changes are driven by changes in magnification effects [38, 41, 42], where the perceived position of the step appears closer when viewing through positive-powered lenses. Collectively, these studies highlight the potential for gait changes in older adults in the presence of uncorrected refractive error or when using different optical corrections and may become more apparent after updating spectacles or following cataract surgery due to changes in magnification.

Research also shows that blurring vision using cataract simulations in healthy older adults resulted in more cautious gait behaviours when stepping down [43]. Furthermore, older adults with cataracts demonstrated slower gait speeds when compared to controls, and showed improvements in gait speeds following cataract surgery [44].

Studies have also shown changes in gait characteristics in individuals with eye conditions affecting central vision. Research has demonstrated slowing of walking speeds and other gait changes with simulated central visual field loss [45]. Early clinical studies of older adults with AMD reported more cautious gait behaviours, particularly walking more slowly on uneven terrain compared to controls [46]. Furthermore, individuals with central vision loss showed slower walking speeds and higher foot clearances than controls when avoiding obstacles [47], as well as a reduction in balance confidence in those with AMD, manifesting in a cautious and controlled landing behaviour during step or stair ascent [48]. Greater severity of central vision loss from AMD, particularly contrast sensitivity, has been associated with slower walking speeds and increased double-support time [26]. Furthermore, studies conducted under low light environments show that older adults with AMD have reduced accuracy and increased variability when performing precision stepping tasks [49], and exhibit much shorter steps (shuffling) when approaching curb edges, and increased double support duration when stepping over curbs [50].

Studies have also shown changes in gait characteristics in individuals with visual field loss. Slower walking speeds and other gait changes were demonstrated in the presence of simulated visual field loss [51], as well as in older adults with visual field loss who had slower walking speeds and more obstacle contacts [52, 53]. Differences in a range of gait characteristics in older adults with glaucoma have been demonstrated compared to controls [54], with recent studies showing that greater severity of visual field loss from glaucoma is linked to slower walking speeds [55, 56].

### Gaze Behaviours

The central and peripheral visual system provides critical information about the environment when walking to inform appropriate locomotor output for safe mobility, such as changing body direction and speed to alter the travel course, altering lower limb trajectories to avoid obstacles and guide safe placement of the feet when navigating complex environments. The more cautious gait strategy of slowing walking speed, mentioned above, allows additional time for individuals with vision loss to acquire and process relevant visual information from within the environment. Similar to the adaptive gait changes, visuomotor stepping strategies are highly context dependent, where more challenging environments require closer coordination of gaze position relative to foot fall locations, often with prolonged gaze durations at these future foot fall locations, but this may be at the expense of reducing time to sufficiently scan ahead for future path planning and hazard detection. For example, research has shown that older adults spend more time fixating on low contrast stepping targets and initiating the leg swing to step onto these targets, when compared to high contrast targets [57]. Research also shows that time-pressured scenarios lead to altered gaze strategies such as shortening fixation durations towards obstacles ahead [58]. While this area of research has been growing due to advances in mobile eye tracking technology, there are challenges given the complex data processing involved.

A large body of research has examined the effect of visual field loss on gaze behaviours when walking. Studies highlight that some inappropriate mobility-related gaze strategies may be adopted by those with vision impairment. In one study, individuals with retinitis pigmentosa demonstrated a more active visual search pattern on the ground nearby, but reduced time spent looking ahead, when compared with controls [59]. Individuals with glaucoma looked earlier at future stepping targets and transferred their gaze away earlier, but at the expense of greater foot-placement errors during a precision stepping task [60], while in another study, individuals with glaucoma tended to direct their gaze closer, with more time spent viewing obstacles compared to controls, yet still made more contact with obstacles [61]. Interestingly, a recent small-scale study of older adults with glaucoma explored the potential for mobility-related gaze behaviour training to rectify these strategies, by training gaze strategies to guide more accurate foot placement and obstacle avoidance [62], showing significant differences in timing of gaze shifts away from targets relative to stepping on them, with overall reductions in foot-placement errors and obstacle collisions.

### Falls

Falls are typically described as unintentional events in which an individual comes to rest on the ground, often resulting from impaired balance, environmental hazards or sensory deficits, including vision impairment. While falls have multi-factorial causes, reduced visual input is an important contributory factor in older adults, with an increased risk of falls and hip fractures associated with reduced distance visual acuity [20, 63–69], contrast sensitivity [19, 20, 70–72], visual field loss [64, 73–76] and absence of gross stereopsis [65]. These associations may result from vision impairment impacting postural control, adaptations to gait, loss of balance confidence, as well as reduced ability to detect trip hazards in the environment. Indeed, home hazard management has been shown to be effective in reducing fall risk in older adults with severe vision impairment [77].

The role of optometric interventions, such as correction of refractive error, on falls risk has been explored, given the association between good visual acuity and reduced falls risk. An early multifactorial randomised controlled trial (RCT) failed to find any effect of a visual intervention involving referral to an eye care provider or general medical practitioner. However, the results of many of these referrals were not reported back, and only 26/101 of those referred had new spectacles or cataract surgery [78]. A more well-controlled RCT that also included provision of an updated spectacle correction as an intervention reported an increase in falls risk [79], with 74% of those receiving a major change in refraction falling at least once, compared with 53% of those who had minor changes, suggesting that adapting to major changes in prescription is an important falls risk factor. Indeed, older people have more difficulty adapting to larger changes in prescription [80], with magnification changes being the most likely explanation for some older adults with updated spectacles experiencing increased falls risk [42].

Early studies reported an increased risk of falls in multifocal lens wearers due to blurring in the lower field [81]. A subsequent RCT demonstrated that prescribing single vision lenses to multifocal wearers who were high-risk fallers reduced fall risk, particularly for those who participated in more outdoor activities [82]. Secondary analysis of this RCT further highlighted the association between increased falls risk and Progressive Addition Lens (PALs) wear [83]. Furthermore, a laboratory-based study demonstrated that incorporating an intermediate add in PALs that allowed good short-term reading, also improved gait parameters compared to full addition PALs, potentially reducing falls risk [84].

Cataract is associated with increased falls and fracture risk [20, 85–87], with one study finding high rates of falls and fall injury in older adults with bilateral cataracts awaiting surgery [87]. While some studies report reductions in fall rates following cataract surgery [32, 88–90], with benefits being greater with second eye surgery [88, 91], others have failed to find reductions in fall rates [92, 93] or reported an increase in injurious falls [92]. These mixed findings reflect the complex interaction between improvements in vision following cataract surgery, adaptation issues that may arise due to large changes in refractive status, such as between first and second eye surgery, and also the potential for declines in functional capacity that may occur during the time period prior to cataract surgery.

In older adults with AMD, studies reported increased risk of falls and fractures [35, 85, 94], with increased fall rates associated with more severe AMD and reduced contrast sensitivity and visual acuity [94].

Glaucoma is associated with increased falls risk and hip fractures [68, 85, 95–97], which are associated with more extensive field loss [98, 99], particularly in the inferior binocular field [100], and in those with a faster rate of field progression [101]. Falls tend to occur more in the home [99], which may be related to poor lighting [99] and higher numbers of hazards in the home environment [102].

### Future Work

Understanding how individuals move and interact with their environment in everyday life is critical for identifying risk factors associated with postural instability and falls. Traditional laboratory-based assessments, while informative, may not fully capture the complexity and variability of real-world conditions.

With the increased availability of wearable technology, data can be collected in natural environments for sustained periods of time, using mobile phone technologies [103], body-mounted sensors [104], and ‘smart shoes’ (technology integrated into the shoe) [105, 106]. These innovations will expand our knowledge of movement profiles in real-world indoor and outdoor settings [107].

In addition, while individuals with vision impairment adopt cautious gait strategies and alter their gaze behaviour, they still exhibit postural instability and increased fall rates. Hence, more research is needed to explore effective interventions to reduce risk in this population. For example, vision impairment causes significant reductions in physical activity [108], with associated development of frailty [109]; thus, further exploration of the impact of exercise or physical training interventions is important. However, current exercise programmes do not necessarily reduce fall risk [110, 111], possibly due to greater exposure to environmental hazard risks despite improved functional capacity. Although there is an increase in training ‘how to fall well’, strategies, including squatting, elbow flexion and martial arts-inspired rolling in order to reduce impact forces during falls [112], more research is needed to assess this approach in older individuals with vision impairment.

## Vision Impairment and Driving

The relationship between vision impairment and driving has been explored over many years, with different types of vision impairment negatively impacting driving. Importantly, more recent studies have improved our understanding of these associations using larger sample sizes, a broader range of outcome measures and more advanced analyses. In this section, we describe the different driving outcome measures available and present the association between vision impairment from common eye conditions and driving performance and safety.

The terms driving performance and safety are used widely in the driving literature and refer to off-road clinic-based assessments, driving performance assessed in a range of settings and driving safety derived from state-recorded crash data. While these measures reflect different aspects of driving and its component skills, they often make interpretation of the vision and driving literature confusing, given that they measure driving from different perspectives [113]. Self-reported measures of performance and crash risk have also been used but are not included here, since they are not a good reflection of actual driving performance [114], or crash risk [115]. Night-time driving presents even greater challenges for drivers with vision impairment due to low light conditions, low contrast hazards and high intensity glare sources and has been explored in some studies included in this review.

*Off-road driving assessments* are used to assess driving skills in a clinical setting. One example is the computer-based Hazard Perception Test (HPT) that assesses the ability to detect potential hazards in videos of real traffic situations filmed from the driver’s perspective [116–120]. Delayed HPT response times are associated with crash involvement [121–123], as well as with increased heavy braking events during real-world driving [124]. More recently, a night-time hazard visibility test (NHVT) has been introduced, which comprises videos of night-time driving scenes, with response times being significantly associated with night-time pedestrian recognition distances in closed-road studies [125]. *Driving performance* has been assessed using driving simulators, closed-road circuits and under open-road conditions. In simulators and closed-road studies, outcome measures can include vehicle control (steering, lane-keeping, speed control) and hazard detection and avoidance, while on-road studies include ratings of performance by trained assessors on a standardised route under in-traffic conditions, which have been shown to be associated with self-reported [126–128], state-recorded [129], and at-fault and near-miss motor vehicle collisions (MVC) in naturalistic driving studies [129]. *Driving safety* is typically defined as the number of MVCs, obtained from state or police authorities or through naturalistic driving studies and is expressed as the rate of MVCs over a given time period or distance driven. At-fault MVCs are considered most useful in understanding the role of vision in driving, with stronger associations with vision impairment when at-fault, rather than when all crashes are considered [130].

### Uncorrected Refractive Error and Driving

Optical blur as a result of uncorrected or under-corrected refractive error is a common cause of reversible vision impairment, with one study reporting that uncorrected refractive error accounted for 80% of drivers whose vision failed to meet the legal limit for driving [131]. However, while some individuals drive with blur, the effects on driving performance and safety remain poorly understood.

In repeated measures studies using off-road assessments, blur delayed hazard response times compared to best corrected vision in younger and older drivers on the daytime HPT [118], as well as on the night-time NHVT [132].

Simulator and closed-road driving studies demonstrated that steering accuracy and lane-keeping were unaffected by high levels of blur [133–135], but that sign recognition and road hazard detection were reduced [136]. Importantly, the effects of blur on closed-road driving were exacerbated under night-time conditions compared to daytime [135], where even low levels of optical blur (0.50 DS) reduced night-time pedestrian recognition [137], as well as recognition of pedestrian walking direction [138]. In simulator studies, neither induced nor uncorrected astigmatism impacted daytime driving performance when compared to best-corrected vision [139, 140]. However, a closed-road study reported that correction of low levels of astigmatism using toric contact lenses improved night-time sign recognition, hazard avoidance and pedestrian recognition distances [141]. The effects of optical blur, in any form, on on-road driving performance or crash risk have not been explored and are an important area of future research.

### Cataract and Driving

Cataract is a common cause of reversible vision impairment that reduces central visual acuity and contrast sensitivity and increases disability glare, negatively impacting high and low-contrast driving-related resolution tasks.

In repeated measures studies using off-road assessments, simulated cataract delayed hazard response times compared to best corrected vision using the daytime HPT [142], as well as the night-time NHVT [132]. One study showed delays in daytime HPT performance in older drivers with actual cataracts compared to those with normal vision, but the effect was not significant [143].

Closed-road studies demonstrated that simulated cataracts significantly reduce driving performance for younger and older drivers, with effects being greatest for sign detection, hazard avoidance and driving speeds compared to best-corrected vision [144–146]. Night-time studies have also reported impaired ability to recognise road signs and hazards [147], as well as pedestrians [148], when driving with simulated cataracts. In terms of older drivers with actual cataracts, daytime closed-road driving performance was significantly worse for drivers with bilateral cataracts compared to age-similar controls [149, 150].

There is mixed evidence regarding the impact of cataract on MVC risk relative to controls. Drivers with cataract in one or both eyes were 2.5 times more likely to have a history of at-fault state-recorded MVCs in the previous 5 years than drivers without cataract [151]. Conversely, two other studies reported no significant difference in MVC risk in those with cataracts compared to controls [115, 152].

Cataract surgery remains the primary intervention for improving vision in individuals with cataracts, with associated positive benefits demonstrated for driving performance and safety. Significant improvements in driving performance were demonstrated following both first and second eye surgery in a simulator [153] and closed-road study [149]. Similarly, a 50% reduction in MVC rates was reported following cataract surgery [154], particularly for first-eye surgery [91, 155], where one study reported a 61% reduction in MVCs for first-eye compared to 23% for second-eye surgery [156]. A simulation model of US data suggested that earlier cataract surgery reduces MVC risk by 21%, with a saving of 16% (surgery costs balanced against crash costs) [157].

### Central Vision Loss and Driving

Eye diseases affecting central vision may impact high-resolution driving tasks, including reading signs, detecting lane markings for steering and vehicle control, hazard detection and making critical gap judgements. AMD is the leading cause of irreversible central vision loss, particularly in older populations and has been the focus of recent driving studies.

In off-road studies, delays in day HPT performance were reported in older drivers with AMD, compared to those with normal vision, but the effect was not significant [143], while another study also showed poorer performance on a driving simulator, including delayed braking response times, poorer lane control and higher rates of simulator crashes [158]. No studies have assessed the performance of drivers with AMD on closed-road circuits.

On-road driving performance of older individuals with AMD has been shown to be impaired compared to drivers without AMD [158, 159]. The larger of these two studies reported that drivers with early and intermediate AMD were less safe than age-matched controls, with driving instructor interventions being significantly higher than for controls, but only for drivers with intermediate rather than early AMD [159].

Several studies have reported the MVC risk of drivers with AMD compared to those without AMD. One study reported lower at-fault MVC risk in those with intermediate AMD compared to controls, but similar MVC risk in those with early and advanced AMD compared to controls [160]. Two other studies also investigated MVC rates, with one showing no significant association of AMD with involvement in any MVC [115] and the other reporting that AMD was not associated with increased injurious MVC risk [152]. The lack of association between AMD and crash risk may be due to reductions in driving exposure in this population, through restricting driving to avoid challenging driving situations.

Some conditions may also cause central vision loss in younger individuals, including congenital eye diseases such as hereditary optic atrophy, ocular albinism or Stargardt’s disease, with a growing body of research exploring the use of spectacle-mounted bioptic telescopes as a driving aid for individuals with moderate central vision loss, if visual acuity does not meet the driving standard. These devices are used by bioptic drivers to observe road signs, signals and traffic conditions in the distance [161], with drivers often restricted to daytime or local driving [162].

Off-road studies show improvements in hazard response times in bioptic drivers when using their telescope compared to without; however, response times were slower than those with normal vision [163], while individuals trained to use bioptic telescopes demonstrated significantly longer sign recognition distances when using their bioptic compared to without [164]. Bioptic drivers have also demonstrated safe on-road driving performance, comparable to controls, albeit with some steering and lane positioning problems [162]. Recent naturalistic studies of bioptic drivers have shown higher rates of hard braking events compared to controls [165], while other studies reported no differences in near-collision rates [166].

Studies on MVC risk of bioptic drivers showed MVC rates that were somewhat higher than age-similar drivers with normal vision from other studies [167]. One study reported higher MVC rates in bioptic drivers with poorer visual acuity and greater visual field loss [167], while another reported higher MVC rates in drivers with a lack of previous driving experience, rather than reduced visual acuity or contrast sensitivity [168]. Interestingly, studies highlight that bioptic drivers spend only a small proportion of driving time (<2%) viewing through the bioptic telescope [161], so these drivers predominantly drive without the telescope, with visual acuity that is worse than driver licensing standards [161].

### Visual Field Loss and Driving

Visual fields are important for the detection of objects in the periphery, judging distance and speed, maintaining lane position and anticipatory judgements; with conditions such as glaucoma and stroke (hemianopia) attracting most interest in terms of their impact on driving.

Off-road studies reported delayed daytime HPT response times in drivers with glaucoma compared to controls, with more extensive field loss in the worse eye associated with longer delays [169]. Driving simulator studies report mixed findings, dependent on the simulator fidelity and outcome measures recorded [170–172], with more recent studies reporting that drivers with moderate/severe defects exhibited longer hazard response times and poorer lane-keeping than controls [173]. A closed-road study also reported poorer overall driving scores and more hazards hit in drivers with glaucoma compared to controls [174].

Drivers with glaucoma also demonstrate poorer overall on-road driving performance [175–177], more instructor interventions [176, 178, 179], and problems with lane-keeping, negotiating curves and anticipatory skills [175, 176], which were exacerbated in complex driving situations [176]. However, one study failed to find differences in driving performance between glaucoma and controls [180]. Many of these studies were limited by small sample sizes, typically 20 or less with a range of field loss [177–180], with only Wood et al. [176] assessing larger numbers of glaucomatous drivers. Evidence regarding the extent of glaucomatous field loss and on-road driving varies [175, 179], with some studies failing to find associations between field loss and unsafe driving performance [178, 180].

Evidence from state-recorded MVC studies is also mixed, reporting MVC rates in drivers with glaucoma to be either similar [96, 152], higher [115], or lower [181] than in controls. In a more recent study [182], at-fault MVC rates were higher in drivers with glaucoma with severe field loss assessed through a novel field test than in controls. Moderate/severe field loss in the worse eye was associated with higher at-fault MVC rates compared to those with no defect in one study [183], while another found severe binocular pattern deviation loss was associated with higher MVC rates [184].

There is also evidence that some drivers with glaucomatous field loss have the capacity to compensate for their loss through eye and head movements. For example, more extensive scanning in glaucoma was associated with faster hazard response times in off-road testing [116, 169], drivers with glaucoma who passed a simulator test had increased scanning eye and head movements than those who failed [185], and in a closed-road study, larger saccadic eye movements were associated with better driving performance in those with glaucoma [174]. One small on-road study also showed that the extent of scanning eye movements rather than field loss was related to driver safety ratings [177]; again, highlighting the potential for compensation, which may explain the mixed findings regarding the impact of glaucoma on driving performance and safety.

Fewer studies have explored driving in those with hemianopic field loss. Early studies involved retrospective reviews of driving assessment records or included different assessors of driving performance and lacked control groups [186, 187]. More recently, simulator studies have demonstrated that individuals with hemianopic field loss have difficulties with lane-keeping [188], or problems detecting hazards appearing in the blind field [189–192], with some drivers exhibiting compensatory head and eye movements allowing detection of hazards in the blind field [191, 192]. More recently, a study of a larger sample of drivers with hemianopia [193] reported that 65% passed the driving assessment, with younger drivers more likely to pass; however, the extent of homonymous loss or side of field loss was not associated with safety.

On-road studies of drivers with hemianopia also reported problems with lane positioning, steering steadiness and gap judgements compared to controls [177, 194]. Despite these difficulties, many drivers were rated safe to drive and appeared to compensate for their field defect by more eye and head movements into their blind field than those rated unsafe [177, 195]. The only study reporting MVC rates in this population found higher rates than controls [196]; however, the study was limited by small numbers.

More studies are required to further evaluate driving in those with different levels of glaucomatous and hemianopic field loss. The finding that some drivers with field loss demonstrate the capacity for safe driving through compensatory head and eye movements, with the extent of their field loss not being associated with their driving ability, raises the possibility of training eye and head movements to enable safe driving.

### Future Work

The findings of this review highlight the advances that have been made over recent years in better understanding the impact of vision disorders on driving performance and safety, but also the need for further studies to facilitate safer driving in this population through interventions and vehicle technologies. Such studies should use driving outcome measures that reflect driving in the real-world, such as naturalistic driving, which objectively measures drivers’ performance over longer periods while they drive in their own vehicle along familiar routes. This approach has previously been challenging because of problems with data acquisition and analysis, however recent advances in videos, sensors, data storage and communication devices have made this more viable. The advantages of this approach are that driving performance can be measured under everyday driving conditions, without having study personnel in the vehicle, allowing assessment of driving before and during crashes, as well as recording at-fault near-misses. However, while a series of large-scale studies have used naturalistic techniques, only a few have explored the relationship between vision and driving [165, 166, 197, 198], which provides an important opportunity to better understand this relationship in the future.

Vehicles with advanced driver assistance systems (ADAS) are commercially available and have the potential to assist drivers with vision impairment with the complexity of the driving task. However, while there is data on how ADAS can reduce collisions in general populations, including older drivers [199], current evidence is limited to self-reported perceptions of ADAS in those with vision impairment [200], and simulator studies exploring the nature of interventions that might assist drivers with vision impairment [201]. How these technologies translate to improvements in safety and in maintaining the mobility of drivers with vision impairment remains unexplored. The possibility that autonomous vehicles might enable individuals with vision impairment to maintain their mobility and independence has also been suggested, and is supported by drivers with vision impairment [202]; however, such vehicles are unlikely to be widely commercially available for several decades.

## Vision Impairment and Audition

Numerous studies have examined the impact of dual vision and hearing impairment, known as dual sensory loss (DSL), on functional outcomes, demonstrating associations with reduced quality of life, impaired activities of daily living, poorer general health, multimorbidity, adverse mental health outcomes, increased risk of falls and greater social isolation [203–205]. However, the primary focus of this section is to summarise the effects of vision impairment or blindness on auditory function. This is consistent with the broader range of functional adaptations and changes associated with vision impairment discussed in the other sections of this review, for example, how vision loss has been associated with reduced accuracy in auditory spatial mapping, particularly when blindness occurs early in life [206, 207]. However, for completeness, some effects of DSL on functional vision are briefly summarised later in this section, although beyond the primary scope of the present review.

Importantly, the last decade has seen substantial interest in the effects of blindness (defined here as complete vision loss or light perception only) and, to some extent, vision impairment, on auditory abilities. Partly motivated from a patient benefit perspective, research has explored how auditory abilities change as a result of blindness and the effect of age of onset of blindness, how these impact daily activities and whether the changes can be harnessed for rehabilitation or training purposes. Research has also provided theoretical insights into how the senses interact to generate accurate representations of the environment, how the loss of one sense influences the functioning of the remaining senses, and how a more reliable sensory modality can calibrate a less precise one.

### In the Event of Vision Impairment or Blindness, What Happens to Auditory Abilities?

Auditory abilities can be assessed using a variety of measures that capture different aspects of hearing. Examples of different abilities include localisation, which is the ability to accurately judge where a sound is coming from (‘how far away is that barking dog?’) or perceiving speech (‘can you understand what is said in background noise?’). What has become apparent is that there is not a general increase or decrease across auditory abilities following vision loss [208], but rather *it depends on the task the participant is asked to perform*. Recently, as studies show evidence of significant changes (for better or worse), the question has expanded as to *why* some auditory abilities get better or worse following vision loss. By examining the pattern of results across numerous studies, some general conclusions can be drawn regarding which abilities are enhanced and which are degraded as a result of vision loss [209].

### Which Hearing Abilities Improve Following Vision Loss?

Research has demonstrated that vision loss leads to significant enhancements for a range of auditory abilities, especially with early-onset blindness (before 5 years of age), and smaller or no significant changes with blindness onset later in life. These include relatively simple spatial tasks, such as the ability to locate the azimuth of a sound source in space, particularly for peripheral sounds located further to the sides of the head that straddle the interaural axis [210]. For moving sounds, early-blind individuals also demonstrate significantly enhanced abilities to determine the direction of sound motion [211]. Enhanced performance by blind people for non-spatial tasks has also been demonstrated, with evidence that blind people have better musical abilities than sighted controls. For example, rhythm-discrimination was enhanced in an early-onset blind group compared to sighted controls [212], while another study reported enhanced judgements for pitch changes in speech and music in early-blind individuals [213].

The ability to process sound echoes is also enhanced following full blindness, extending to the use of echolocation in humans, a form of biological sonar, similar to that used by bats and dolphins. Echolocating involves making sounds such as mouth clicks and listening to the returning echoes to detect silent objects, with early-blind participants being significantly better than blindfolded sighted controls at safely detecting and moving around a silent obstacle using echolocation [214]. Studies have also indicated that echolocation has real-life benefits outside the laboratory, including improvements in people’s mobility, independence and well-being [215]. Examples include helping blind people get their bearings and pinpointing their location, finding gaps between parked cars, walking in a straight line and avoiding banging their heads against cupboard doors that have been left open. Agrawal et al. [216] suggested that blind participants who self-reported higher echolocation skills also self-reported higher perceived sound localisation capabilities.

### Which Hearing Abilities get Worse Following Vision Loss?

Blindness does not always confer benefits for processing sound, with some studies reporting that, for certain auditory tasks, blindness leads to significantly degraded abilities. Blindness causes difficulties with more complex spatial tasks, such as judging the position of sounds in space relative to each other (allocentric representations of sound sources in space). In one study, groups of blind children and blindfolded sighted children were presented with three sounds in sequence, the first and the third at −25° and +25°, with the second sound at a randomised location between these two, and participants had to judge whether the second sound was closer to the first or the third sound [217]. The blind group performed significantly worse than the sighted group, reproducing previous findings with adult participants [207]. As blind people rely heavily on sound to orient themselves in different places, difficulties with forming spatial maps based on the relationship between sound locations may result in problems when navigating through the environment. This is reinforced by findings showing that early-blind participants show significant deficits when reproducing sound movement either by pointing [218] or walking [219] and riskier road crossing decisions (based on a minimum of 4 s elapsing between pressing a response button and vehicle arrival) compared to sighted controls [220]. However, rehabilitative work using sound and self-controlled movement has shown promise in overcoming these deficits. Wearing a sound-producing bracelet that provided audio feedback about body movements was shown to significantly aid blind children when building a sense of their surrounding space [221].

Another auditory ability that is degraded following blindness is judging how far away sounds are (absolute distance judgement). In one study, blind participants and blindfolded sighted controls were presented with speech, music, or noise sounds at virtual distances between 1.2 and 13.8 m, simulated within rooms having normal echoic characteristics (such as an office) or without echoes [222]. Regardless of the room environment or sound type presented, blind participants overestimated the distance to close sources and underestimated the distance to far sound sources. Effectively, the loss of vision results in a ‘compressed’ representation of the auditory world, due to the lack of detailed spatial information afforded by vision. This has consequences for safety issues, such as hearing an approaching vehicle and having time to plan to avoid collisions.

### Why do Some Hearing Abilities Become Enhanced and Others get Degraded Following Vision Loss?

Auditory abilities where vision does not play a role, for example, non-spatial tasks such as musical judgements, are often enhanced for blind individuals compared to sighted controls. Brain imaging research has shown that in the event of vision loss, especially early-onset blindness, brain areas that would otherwise process vision, such as the occipital cortex, might be recruited by the auditory system and start processing sound instead [208], thereby increasing the resources available to process sound, possibly underling enhanced performance. The training associated with additional reliance on sound resulting from blindness may also contribute to enhanced auditory abilities [209].

However, more complex spatial tasks where vision contributes by helping to calibrate auditory mapping of the world are often impaired following blindness. These include absolute distance tasks (‘how far away is that sound’) and distance bisection tasks (‘is that second sound you heard closer to the first or the third sound?’), suggesting that vision is necessary to build complex spatial representations of the world. Blindness early in life leads to certain spatial deficits, such as mapping the relationship between sound positions in space, or judging the distance to objects making sounds. Early intervention is therefore preferable, and one way that spatial auditory abilities in blind individuals might be improved is through effective use of technology; however, assistive devices need to be specifically designed to achieve this goal.

### Functional Changes with Dual Sensory Loss

Dual sensory loss often occurs with increasing age and is a relatively common condition that can significantly impact daily activities [223], and can lead to difficulties including localising sound sources, speech recognition and wayfinding [224]. Vision offers important speech-reading cues during conversation, spatial layout information, as well as alerting signals, and DSL can significantly impact independent travel involving safely navigating complex surroundings and using sound to localise moving traffic. While hearing aids have been found to have high utility for understanding speech, they have been reported to be less effective for localising sounds that help situational awareness during independent travel, especially for people with blindness and hearing loss [225]. For individuals with DSL who are unable to use visual cues to compensate for difficulties localising sound sources, audiologists might recommend certain models of hearing aid or adjust the settings of existing hearing aids that help to identify the direction of sound sources [226].

### Future Work

Although the effects of full blindness have received attention, there has been less research regarding the effects of partial visual loss (defined here as remaining vision that is better than light perception, where vision information is present but degraded) on auditory abilities. This may be due to difficulties in maintaining experimental control given the heterogeneity of participants with vision impairment, e.g., those with peripheral or central loss or scotomas and with different severities. Blind and partially sighted individuals differ in that partially sighted individuals develop an auditory spatial map supported by both remaining vision as well as audition, have less plasticity for visual brain areas, which still receive some visual input and may demonstrate abnormal orienting behaviours [227]. Although some studies show no significant effects of partial vision loss on auditory abilities, such as locating the vertical position of a static sound source [228], or distance discrimination [229], there is evidence that greater severity of visual loss leads to larger auditory judgements of distance [230], and that partial visual loss leads to lower accuracy when judging the left-right position (the azimuth) of sound sources [227]. There is, therefore, a real need for more research in this area.

As vision plays a central role in spatial awareness and navigation, its loss necessitates greater reliance on other sensory modalities. Consequently, understanding how auditory and tactile systems can compensate for visual deficits has also become an important focus in rehabilitation research. Auditory training has shown promise as an effective means of rehabilitation for those who have lost their vision [215]. However, further work is needed to design auditory training programmes for more challenging situations, as multiple sound sources, background noise and reverberation are commonplace in everyday environments. In addition, the effectiveness of echolocation for safe navigation, which to date has generally occurred in quiet, controlled locations [214], needs to be assessed in busier, noisier environments. The role of assistive technology for the visually impaired is also a focus of future research. For example, in older children and adults, Sensory Substitution Devices are electronic travel aids for the blind that provide information about the world through touch or sound information. These can be effective for navigating and detecting and avoiding obstacles [231, 232]; however, user acceptance of assistive technologies is often low [233]. Future research needs to investigate ways of making technology for the blind more accessible, with a focus on rehabilitation that increases brain plasticity [233], and with input at the design stage from blind individuals to increase user acceptance and decrease complexity [215, 231, 232].

## Summary

This review highlights the importance of understanding the functional impacts of vision impairment, particularly in older adults, as age-related vision loss increases the risk of falls, mobility limitations and reduces independence. Research has highlighted how various types and severities of vision impairment, ranging from reduced visual acuity, contrast sensitivity and visual field loss to conditions including AMD, glaucoma and cataract, affect balance, gait and fall risk, with individuals often adopting cautious movement and gaze strategies to compensate. Vision impairment also affects driving performance and safety, with deficits in central or peripheral vision, cataract or uncorrected refractive errors influencing hazard detection, lane-keeping and crash risk, though compensatory strategies and interventions such as cataract surgery, bioptic telescopes or emerging driver-assist technologies show promise. Finally, vision loss alters auditory abilities with improvements in some functions, particularly non-spatial tasks and echolocation, while complex spatial hearing and distance judgements are often degraded, highlighting the role of vision in calibrating auditory perception. Emerging approaches using wearable sensors, naturalistic assessments and auditory training aim to better understand and mitigate these functional limitations, supporting independence and quality of life for those with vision impairment. This review highlights opportunities for future research on the effect of partial vision on auditory abilities and to explore and develop more effective interventions to support safety, mobility and functional independence in people with vision impairment.

## Data Availability

No datasets were generated or analysed during the current study.
